# The European respiratory society guideline for adult bronchiectasis 2025: summary and implementation guide for primary care

**DOI:** 10.1038/s41533-026-00528-z

**Published:** 2026-06-08

**Authors:** Fiona Mosgrove, Janwillem WH Kocks, Eliza Kompatsiari, Alan Timothy, Tanja Hedberg, Merete B. Long, Stefano Aliberti, James D. Chalmers

**Affiliations:** 1 School of Medicine, University of Dundee, Ninewells Hospital and Medical School, Dundee, UK; 2 Portlethen Medical Centre, Portlethen, Aberdeenshire, UK; 3 General Practitioners Research Institute, Groningen, The Netherlands. Observational and Pragmatic Research Institute, Singapore. Groningen Research Institute Asthma and COPD (GRIAC), University of Groningen. University Medical Center Groningen, Groningen, The Netherlands. Dept of Pulmonology, University of Groningen. University Medical Center Groningen, Groningen, The Netherlands; 4 European Lung Foundation / EMBARC Patient Advisory Group, Sheffield, UK; 5 IRCCS Humanitas Research Hospital, Respiratory Unit, Rozzano, MI Italy; 6 Department of Biomedical Sciences, Humanitas University, Pieve Emanuele, MI Italy; 7 Radcliffe Department of Medicine, University of Oxford, Oxford, UK

**Keywords:** Diseases, Health care, Medical research, Microbiology

## Abstract

Bronchiectasis is a common chronic respiratory disease with rising prevalence, hospitalisation rates, and mortality. It is estimated to affect approximately 1 in 200 adults, imposing a substantial symptom burden and significant healthcare costs, largely driven by exacerbations. Although diagnosis and long-term management are usually led by respiratory specialists, most patient care interactions occur in primary care, including the management of multimorbidity and acute exacerbations. In December 2025, the European Respiratory Society (ERS) published updated global clinical practice guidelines for adult bronchiectasis. This article summarises the 2025 ERS recommendations with a specific focus on their implementation in primary care practice. Key priorities include improving early recognition and reducing diagnostic delay, undertaking standardised investigations to identify underlying causes and treatable traits, and recognising features associated with poor outcomes that warrant specialist referral. The guidance emphasises the importance of sputum microbiology, including testing for non-tuberculous mycobacteria, and targeted blood investigations such as immunoglobulins and allergic bronchopulmonary aspergillosis serology. Core management strategies relevant to primary care are reviewed, including airway clearance techniques, pulmonary rehabilitation, and evidence-based use of inhaled therapies. The article outlines best practice for the management of acute exacerbations, highlights differences from asthma and COPD care, and clarifies the limited role of inhaled corticosteroids in bronchiectasis. The identification and monitoring of patients who may benefit from long-term antibiotic therapy, including those with *Pseudomonas aeruginosa* infection, are also discussed. By translating specialist guideline recommendations into a primary care context, this summary aims to support timely diagnosis, optimise ongoing management, and improve outcomes for adults with bronchiectasis.

## Introduction

Bronchiectasis is the third most common respiratory disease behind asthma and COPD worldwide^1^. Patients typically present with a chronic productive cough with mucopurulent sputum and recurrent chest infections^2^. The most recent estimates suggest approximately 1 in 200 adults have bronchiectasis, meaning an average UK general practice with a patient population of 15,000 patients might care for 70 patients with bronchiectasis, though increasing rates of diagnosis mean that this is likely to be an underestimate^1,3,4^. The rates of hospitalisation and mortality from bronchiectasis are rising rapidly by approximately 4% per year^1^. Patients’ experience a high symptom burden with cough and sputum production contributing to fatigue and tiredness in addition to anxiety and depression with haemoptysis and weight loss occurring in some individuals^5,6^. The healthcare costs of the disease are high, vary geographically and are largely driven by care provided for patients during pulmonary exacerbations.

Bronchiectasis is a highly heterogeneous condition with multiple possible underlying aetiologies, and its management can be complex^7^. In most countries, management is undertaken by respiratory specialists in secondary or tertiary care centres. However, most healthcare interactions for adult patients with bronchiectasis are with primary care professionals. Patients with bronchiectasis are typically multimorbid with other conditions including cardiovascular disease, depression, osteoporosis, asthma, chronic obstructive pulmonary disease (COPD), and gastro-oesophageal reflux disease (GORD)^8^. Patient centred management of multi-morbidity often sits best with primary care professionals. In addition, most exacerbations are initially, if not entirely, managed in primary care even for patients under the care of a specialist respiratory physician.

**Fig. 1:**
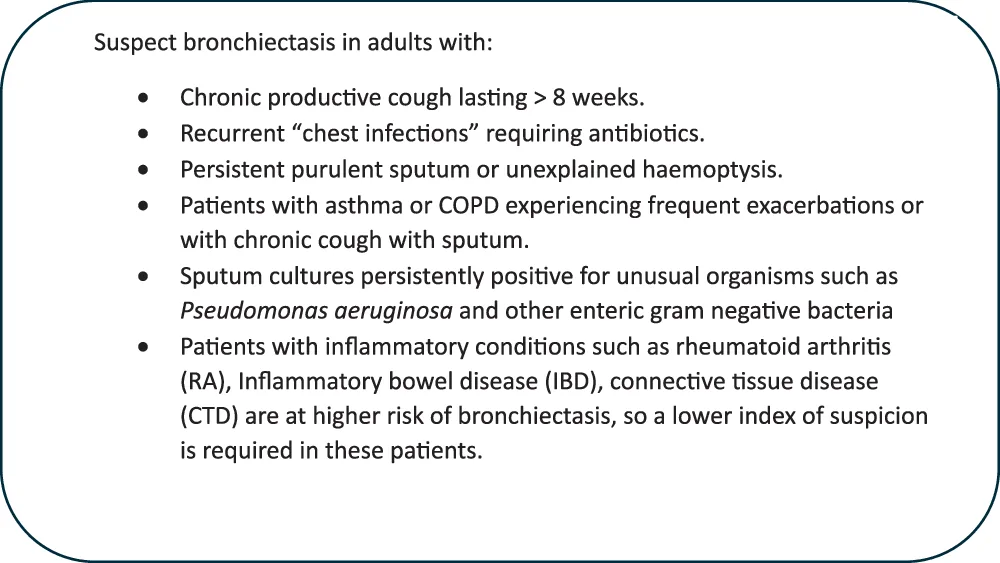
Common reasons to suspect bronchiectasis.

Patients with bronchiectasis experience a significant delay in diagnosis, with 25% of patients waiting more than 10 years for a diagnosis^9,10^. Misdiagnosis of asthma, COPD and bronchitis is common^11^. Symptoms of bronchiectasis overlap significantly with those of other airway diseases and there is a complex interaction between asthma, COPD and bronchiectasis. Asthma and COPD can be co-morbid with bronchiectasis or can be the cause of it, particularly when these diseases are poorly controlled. As a result of this complexity, patients are often diagnosed with asthma or COPD before bronchiectasis is recognised. The key reasons to suspect bronchiectasis are outlined in Fig. 1 and patients presenting with these should have a high resolution computed tomography (HRCT) scan arranged

In December 2025, the European Respiratory Society issued new global guidelines for the management of adult patients with bronchiectasis^7^. The aim of this article is to summarise those recommendations as they apply to healthcare professionals working in primary care. The article only addresses bronchiectasis not related to cystic fibrosis, the treatment for which is different and beyond the scope of this article.

In the following sections we go through the key components of management and recommendations in the guidelines highlighting the guideline recommendations and the implications for primary care.

## Underlying causes of bronchiectasis: implications for primary care

### Recommendations

**Management of patients with bronchiectasis should include standardized testing to identify the underlying cause of bronchiectasis, to evaluate disease severity and activity as well as risk of poor outcome, and to identify co-morbidities and associated treatable traits (Strong recommendation, moderate certainty of evidence stemming from narrative review of the evidence)**

### Diagnosing bronchiectasis

Bronchiectasis is diagnosed by the finding of dilated bronchi on high resolution computed tomography (HRCT) scan of the chest^12^. In most areas, primary care does not have direct access to HRCT. As a result, diagnosis is often made after a referral to secondary care, where patients are assessed and a scan is then requested. Figure 1. shows common presenting symptoms to be aware of and patient groups that are at higher risk of bronchiectasis where a higher index of suspicion is wise. Patients with a label of either asthma or COPD with frequent exacerbations or ongoing productive cough with purulent sputum despite optimal treatment are a key group to look out for in primary care. Patients sometimes have a historic label of asthma or COPD that is not backed up by objective testing or has never responded to the relevant treatment. It is worth re-evaluating these diagnoses, particularly if the clinical picture is of cough with discoloured sputum, as most patients with asthma and COPD in primary care have not had a CT scan performed, and these are patients that could well have bronchiectasis as the underlying diagnosis. Certain inflammatory conditions are associated with bronchiectasis and increase the likelihood of a patient developing bronchiectasis. 5–10% of patients with rheumatoid arthritis, inflammatory bowel disease and other connective tissue diseases have bronchiectasis depending on the definitions used^13^.

**Fig. 2:**
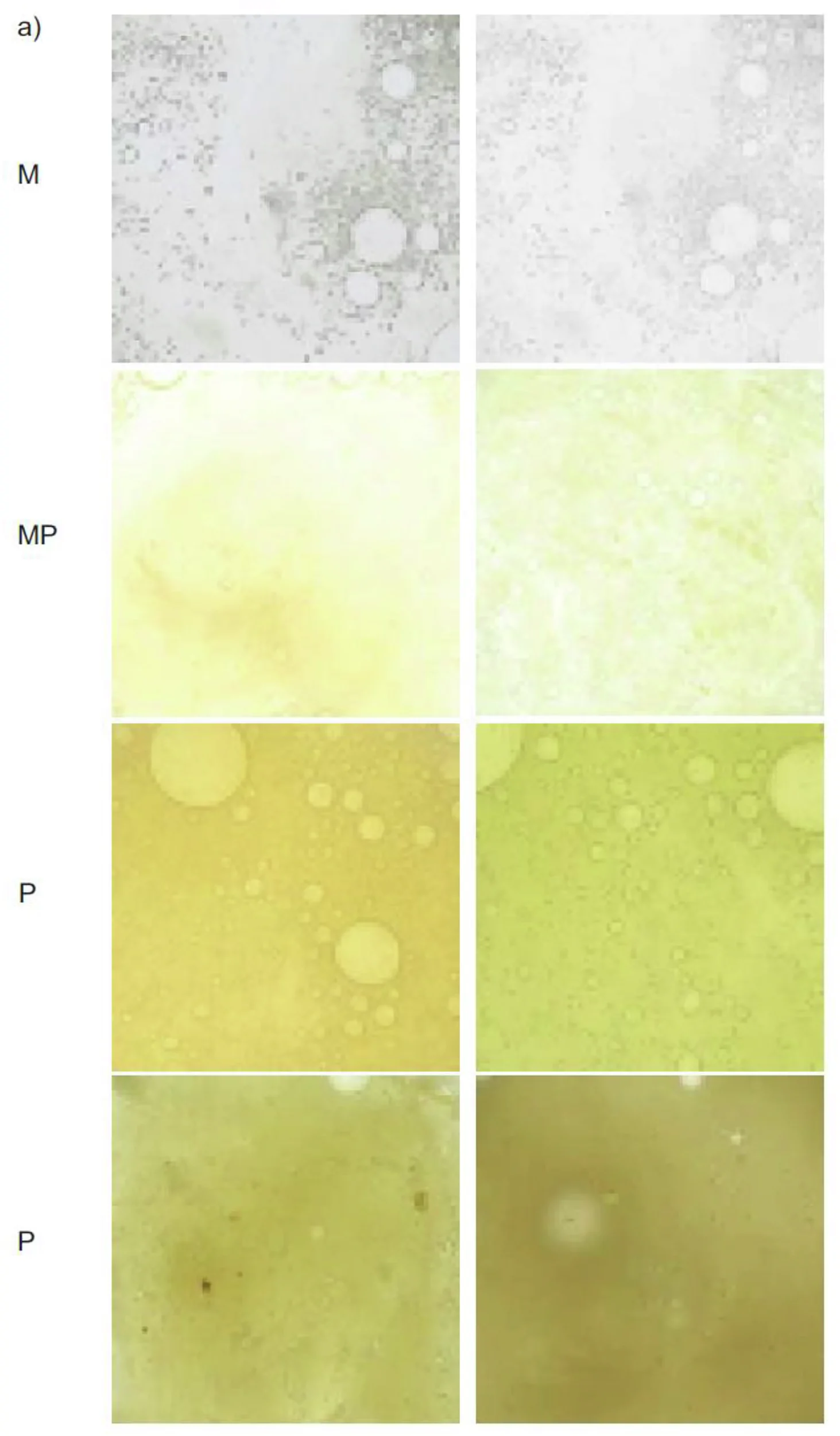
sputum colour chart indicating mucopurulent (MP) and purulent(P) sputum^54^.

Certain clinical features in bronchiectasis patients are linked to a higher risk of poor outcomes. These features should be highlighted in referral letters for new patients with suspected bronchiectasis. If patients with an established diagnosis start to display any of these features, urgent re-referral to secondary care is advisable. These features include:

- 2 or more exacerbations per year
- severe daily symptoms of cough and sputum production
- purulent sputum production when not exacerbating(see Fig. 2)
- patients with high-risk causes (RA, primary ciliary dyskinesia (a genetic condition responsible for up to 10% of cases of bronchiectasis, often starting in childhood), COPD, non tuberculous mycobacterial infection, allergic bronchopulmonary aspergillosis)
- Patients with high risk pathogens in sputum (*Pseudomonas aeruginosa*, NTM)^2,6,7^.

### Aetiological testing

Management of the underlying aetiology of bronchiectasis is essential to good disease control. Aetiological tests are usually performed once the diagnosis is confirmed and this is usually done in secondary care. Bronchiectasis is described as idiopathic in approximately 20–40% of cases, with significant variability between centres. Some of this variability can be explained by heterogeneity in aetiological testing in bronchiectasis patients^14^. The standard tests recommended for bronchiectasis are: Full blood count, immunoglobulins, serum electrophoresis, allergic bronchopulmonary aspergillosis (ABPA) serology, and sputum for culture including non-tuberculous mycobacterium (NTM)^7^. This panel of testing should be performed in all patients with confirmed bronchiectasis. Further testing is often performed if initial tests are negative and the history suggests a rarer cause such as primary ciliary dyskinesia (PCD) or cystic fibrosis (CF). The key aetiological tests and relevant clinical features are highlighted in Table 1. The basic tests are accessible to primary care even if in most healthcare settings bronchiectasis is diagnosed and evaluated in secondary care.

**Table 1:** Investigations to consider when exploring aetiology of bronchiectasis.

Underlying cause / issue	Clinical features that may raise suspicion	Test to request	Rationale
Non-tuberculous mycobacteria (NTM)	Persistent symptoms despite antibiotics; recurrent exacerbations; classically tall, thin patients; may have non-productive cough, weight loss and constitutional symptoms (fever, malaise, anorexia)	Sputum for acid-fast bacilli (AFB) culture	NTM are not detected on routine sputum culture; delayed diagnosis is common and treatment is prolonged and complex
Allergic bronchopulmonary aspergillosis (ABPA)	Very thick, sticky sputum (often described as “glue-like” or in solid plugs); difficulty expectorating; co-existing asthma or COPD	Total IgE, *Aspergillus*-specific IgE and IgG, Full blood count (for eosinophilia)	Identifies ABPA, a treatable cause of bronchiectasis requiring immunomodulatory therapy rather than antibiotics alone
Primary immunodeficiency	Recurrent or severe infections; early onset disease	Immunoglobulins (IgG, IgA, IgM)	Detects antibody deficiency, allowing targeted management to reduce future infections
Secondary immunodeficiency / haematological disorders	Recurrent infections; anaemia or other dyscrasias	Serum protein electrophoresis and immunoglobulins	Immune deficiency or dysfunction contributing to bronchiectasis
Systemic inflammatory disease (e.g. rheumatoid arthritis, inflammatory bowel disease, connective tissue disease)	Known inflammatory disease; multisystem symptoms; inflammatory flares	Careful clinical history±inflammatory markers	Identifies bronchiectasis associated with systemic disease, where deterioration may not always be infective

Sputum culture should be performed in all patients with confirmed bronchiectasis. This may not have been done in patients initially diagnosed with asthma or COPD, as sputum culture is not routinely recommended for these conditions. Up to 70% of patients are chronically infected with pathogens meaning sputum cultures will be positive even when they feel “well”. Treating these patients with relevant antibiotics often makes no difference to their symptoms. Spirometry is not specifically recommended as an aetiological test but is often performed in primary care in patients presenting with respiratory symptoms. There is no specific spirometry pattern associated with bronchiectasis; patients may have normal spirometry, a restrictive pattern, an obstructive pattern, or a mixed restrictive and obstructive pattern. However, an obstructive pattern is the most common, and FEV% predicted forms part of bronchiectasis severity assessment^15–17^.

ABPA is the development of an allergy to *Aspergillus*, a common environmental mould^18^. It can be co-morbid with asthma and COPD and is the cause of bronchiectasis in approximately 10 percent of patients, with geographical variation^18–20^. It presents with very viscous sputum which is extremely difficult for patients to expectorate. Treatment is started in secondary care, usually with high dose prednisolone, and/or antifungals, and/or with monoclonal antibodies which are only used under specialist guidance^18^ Identifying ABPA or suspected ABPA as potential cause of bronchiectasis in primary care can lead to a quicker assessment of the patient in secondary care and prompt initiation of treatment which may improve outcomes^18^.

Non-tuberculous mycobacteria (NTM) are a collection of organisms present in soil and water that do not usually act as respiratory pathogens^21^. In the presence of structural lung disease or local immunosuppression, as is the case in airway disease such as COPD and bronchiectasis, non-tuberculous mycobacterial pulmonary disease (NTM-PD) can develop. Mycobacterium avium complex is by far the most common organism found causing infection in people with bronchiectasis. The presentation may be indistinguishable from bronchiectasis without NTM, but classically patients are more likely to be tall thin females with a non-productive cough^22^. Treatment is with a combination of antibiotics for an extended period, often 18 months to 2 years^23^. Unless sputum is specifically sent for NTM culture (acid fast bacilli), then it cannot be diagnosed. As a result, NTM-PD is often diagnosed late, when disease is more challenging to treat. Sending sputum for culture for acid-fast bacilli is recommended in all patients with confirmed bronchiectasis at diagnosis and is particularly relevant in patients with persistent symptoms that do not improve or resolve with antibiotics. It is also advisable to send sputum for NTM culture prior to initiation of long term macrolides, as NTM resistance can arise if patients are given macrolides alone.

### Managing multimorbidity

Multimorbidity presents a complex challenge and can have profound effects on patient quality of life, psychosocial functioning and ability to self-manage. Primary care professionals are well placed to work with patients to identify and manage relevant co-morbidities. Patients with bronchiectasis have an average of 3–4 co-morbidities^8,24^, many of which are non-pulmonary. Like patients with COPD, patients with bronchiectasis are at increased risk of cardiovascular disease. Osteoporosis is also common in bronchiectasis. Gastro-oesophageal reflux, rhinosinusitis, and anxiety/depression are also frequently reported.

Part of the initial assessment of the patient in primary care should include:

- evaluation of relevant co-morbid conditions,
- cardiovascular risk assessment
- osteoporosis risk assessment
- Identification of important treatable traits such as rhinosinusitis, reflux, and mood disorders.
- Optimisation of co-existing airway disease

### Airway clearance and mucoactive drugs in primary care

**Recommendation**

**We recommend that patients with bronchiectasis should be taught airway clearance techniques (strong recommendation, very low certainty of evidence)**

**We suggest offering mucoactive treatments to patients with bronchiectasis where airway clearance has failed to control symptoms (conditional recommendation, very low certainty of evidence)**

**We suggest not to offer recombinant DNAse to patients with bronchiectasis (conditional recommendation, very low certainty of evidence)**

Airway clearance techniques are vital for bronchiectasis patients and are best taught by a specialist respiratory physiotherapist^25^. There are a variety of techniques which can be used, and each patient will benefit from an individual assessment to explore which techniques work best for them and if any adjuncts are required. Access to respiratory physiotherapy directly from primary care is often not available and access in general, even from secondary care is often poor. Newly diagnosed patients should be referred for detailed physiotherapy assessment by their respiratory specialist, where physiotherapy is available. Adherence by both clinicians and patients to airway clearance is poor. Less than 50% of patients in Europe have been shown how to perform airway clearance techniques, and data suggests only around half of patients practice these exercises regularly^26,27^. Individualised assessment is critical for patients as there is no single airway clearance technique that suits all patients, and finding the technique that is most effective for each patient is likely to improve their concordance with this important component of disease management. Primary care can have a role in encouraging patients to perform airway clearance regularly, particularly in those with frequent exacerbations or a heavy symptom burden. Encouraging patients to maintain adequate hydration is a key component of airway clearance, as dehydration is associated with thicker, more viscous mucus^24^.

Patients struggling to expectorate sputum who have never learned airway clearance, or whose technique has deteriorated over time should be referred to specialist care or directly to a specialist respiratory physiotherapist. In resource limited settings where access to specialised respiratory physiotherapy is not available, primary care professionals could direct patients to online or written materials on airway clearance^7^ such as the European Lung Foundation patient priorities websites^7,28,29^. These resources are useful but are not a replacement for professional physiotherapy input. In some centres, physiotherapists are involved in training nurses to deliver education to patients on simple airway clearance techniques, to improve patient access to these. Initial results were positive, with an overall improvement in quality of life reported by patients^30^.

Mucoactive drugs are often initiated in secondary care and include oral agents such as carbocisteine and N-acetylcysteine, and inhaled therapies such as hypertonic saline. These treatments aim to improve mucus clearance by reducing sputum viscosity or increasing airway hydration, thereby facilitating expectoration. They are an adjunct to airway clearance measures, not a replacement for these. Although typically initiated in secondary care, primary care clinicians may be involved in managing side effects, reviewing ongoing benefit, or supporting treatment decisions.

Carbocisteine is thought to modify mucus composition by reducing viscosity and improving mucociliary clearance. However, recent evidence has not demonstrated clear benefit in bronchiectasis, and discontinuation may be considered in patients who do not perceive symptomatic improvement. In patients who have been taking carbocisteine for prolonged periods without clear benefit, a supervised trial of cessation may be appropriate^31^.

N-acetylcysteine acts as a mucolytic by disrupting disulphide bonds within mucus glycoproteins, reducing sputum viscosity and aiding clearance^32^. Evidence for its effectiveness in bronchiectasis is mixed, and ongoing benefit should be reviewed on an individual basis.

Hypertonic saline improves airway clearance by increasing airway surface liquid, enhancing mucus hydration and stimulating cough. The most common adverse effect is bronchospasm, which may occur unpredictably, even after prolonged use. Symptoms include chest tightness, breathlessness, or worsening cough. Pre-treatment with a bronchodilator, such as nebulised salbutamol 2.5 mg, may reduce this risk. If symptoms persist, hypertonic saline should be discontinued or replaced with a lower tonicity solution^7^.

### Pulmonary rehabilitation

**Recommendation**

**We recommend that patients with breathlessness and/or impaired exercise capacity should be offered pulmonary rehabilitation (strong recommendation, very low certainty of evidence)**

Breathlessness and exercise limitation are common symptoms of bronchiectasis with a major impact on patients’ quality of life^33^. Patients with bronchiectasis benefit from pulmonary rehabilitation at least as much as patients with COPD and this is the basis for a strong recommendation in favour of pulmonary rehabilitation in the guidelines^7,34^. Pulmonary rehabilitation should be considered a core component of bronchiectasis care, as it is in COPD. Patients with bronchiectasis who have noticeable breathlessness that affects daily life are good candidates for referral to pulmonary rehabilitation services. Intervening before breathlessness is very severe is usually beneficial. However, patients who are more severely breathless can still benefit. Pulmonary rehabilitation is generally underutilised and many patients who are referred do not attend classes. It is known that geographical location and infrastructure affect patients’ ability to attend as does their social context including social support and beliefs about their illness and the likely efficacy of PR^7^. If clinicians can help patients to see that the classes will help improve self-efficacy and allow development of useful skills to help self-management, this may help improve participation.

### Long term antibiotic treatments: impacts on primary care

**We recommend offering long term inhaled antibiotics to patients at high risk of exacerbations and chronic infection with** ***Pseudomonas aeruginosa*** **(Strong recommendation, moderate certainty of evidence)**

**We suggest offering long term inhaled antibiotics to patients at high risk of exacerbations and chronic infection with pathogens other than** ***Pseudomonas aeruginosa*** **(Conditional recommendation, moderate certainty of evidence)**

***Recommendation***

**We recommend offering long-term macrolides to patients at high risk of exacerbations despite standard care (Strong recommendation, moderate certainty of evidence)**

**Recommendation**

**The panel suggests NOT to offer long-term non-macrolide oral antibiotics as a first line treatment to adult patients with bronchiectasis and a high risk of exacerbations (conditional recommendation against the intervention, very low certainty of evidence)**.

Deciding which patients at high risk of exacerbations will benefit from long term preventative treatments is nuanced and complex. These decisions are best taken by specialists with expertise in the management of patients with bronchiectasis, usually in secondary care. Preventive treatments include long term macrolides and inhaled antibiotics^35^. There are three major implications of these recommendations for primary care clinicians. The first is identifying patients with frequent exacerbations or a high symptom burden who should be referred for consideration of these treatments. The second is managing the possible side effects associated with them^7^. The third implication is that macrolide prescribing in bronchiectasis is off-label and this can result in some questions over who should prescribe macrolides and which budget the funding should come from.

Treatment with long-term macrolides is usually with azithromycin at doses varying from 250 mg three times a week to 250 mg daily^36^. Azithromycin acts as a powerful anti-inflammatory agent in the airways in addition to having anti-microbial activity. Sometimes clarithromycin or erythromycin is used in its place due to clinician preference or drug availability. Macrolides are highly effective in reducing exacerbations rates in bronchiectasis, by 50%. They also improve quality of life by reducing the production of sputum but, they have some important side effects to be aware of^36^. Up to 1 in 5 patients will experience gastrointestinal adverse effects such as diarrhoea, due to the prokinetic effects of macrolides, which may lead to discontinuation. This is worse with higher doses e.g. 250 mg daily vs three times per week, and alternative day dosing is recommended by the guidelines as this may reduce side effects. As use of macrolides grows, some patients on these may be discharged to primary care after successful treatment for a year or more, if they are stable.

Tinnitus and hearing loss are rare but recognised side effects of macrolide therapy which are reversible if macrolide therapy is discontinued. Patients on macrolides presenting with a new onset of reduction in hearing or tinnitus should have their macrolide stopped and the tinnitus investigated, usually by audiology^7^. Macrolides can prolong the QT interval, and patients will routinely have an ECG prior to starting treatment to make sure their QT interval is normal. If patients are subsequently started on other medications which may prolong the QT interval, close monitoring of ECG is advisable. If a patient taking macrolide therapy does exacerbate, it is safe for them to continue their macrolide treatment in addition to antibiotic treatment for the exacerbation, unless the antibiotic being used also tends to prolong the QT interval (the most common examples being ciprofloxacin or another macrolide).

Macrolides are recognised to cause idiosyncratic drug-induced liver injury, but clinically significant liver toxicity is uncommon and the incidence of this with long-term prophylaxis is poorly quantified^37,38^. When clinically significant liver dysfunction occurs, it tends to be early after starting treatment^38^. As a result, many clinicians perform baseline testing of liver function and repeat these after 2–4 weeks.

For inhaled antibiotics, the most common side effect is bronchospasm presenting with chest tightness, breathlessness or coughing often immediately after inhalation of the drug^39^. The guidelines emphasise that patients should be encouraged to perform airway clearance and take bronchodilators prior to taking their inhaled antibiotic to increase tolerability, safety and efficacy of these^7^. Patients initiated on inhaled antibiotics usually have a trial of these performed in secondary care, to assess tolerability, prior to initiation.

2026 has seen the licensing of the first bronchiectasis specific treatment, the Dipeptidyl peptidase 1 (DPP-1) inhibitor brensocatib^40^, with similar molecules in clinical trials^41^. It is likely that these medications will be initiated in secondary care, and the main side effect to be aware of is hyperkeratosis (thickening of the skin), which is usually mild and affects the hands and feet.

### Pseudomonas aeruginosa eradication

**Recommendation**

**We suggest offering eradication treatment to patients with a new isolation of** ***Pseudomonas aeruginosa*** **(conditional recommendation, very low certainty of evidence)**

*Pseudomonas aeruginosa* is an important pathogen in bronchiectasis^42^. In Europe, it is present in 22% of all patients with bronchiectasis who have sputum samples sent for culture, with metagenomics studies indicating the prevalence in Southern Europe is as high as 33%^16,43^. Isolation of Pseudomonas is a predictor of poor outcomes in patients with bronchiectasis and is associated with a 3-fold increase in mortality and a 7-fold increase in hospitalisations^44^. *Pseudomonas* is well adapted to survive in the airways and, once established, it can be extremely difficult to eradicate. This is partly due to its ability to form biofilms which protect it from antibiotics^45^.

Sputum culture results that report scanty or profuse growth of *Pseudomonas*, without explicitly confirming infection, are sometimes overlooked or considered clinically irrelevant by microbiology laboratories and by clinicians. However, this is an error, and any isolation of *Pseudomonas* should prompt a repeat culture and immediate discussion with secondary care colleagues. A wait and see approach is never appropriate as isolation of Pseudomonas indicates a turning point in the disease which needs urgent, structured management. Eradication is usually attempted and it is likely that the closer to first isolation of pseudomonas this is attempted, the more likely the patient will benefit.

Recommended regimens include oral ciprofloxacin (500–750 mg BD for 2 weeks); however, intravenous or inhaled antibiotics are often also required. It is for this reason that management should be discussed with secondary care colleagues at the earliest stage, as culture is being repeated, and before initiation of ciprofloxacin^7,42,46^. Though other organisms, such as *Haemophilus influenzae* can be identified in sputum, eradication of these is not usually attempted.

### Inhaled corticosteroids

**Recommendation**

**We suggest not to offer long term inhaled corticosteroids to patients with bronchiectasis who do not have coexisting COPD or asthma (conditional recommendation, low certainty of evidence)**

Inhaled corticosteroids (ICS) are a common prescription in primary care for patients with respiratory symptoms. They are the cornerstone of management of asthma and have a role in exacerbation reduction in COPD^47^. It can be difficult to stop ICS once it has been started and if patients fail to respond to low dose treatment, they are often moved to higher strength ICS, particularly in asthma. It is important to remember that ICS treatment increases the risk of pneumonia, may promote bacterial infection of the airways which is a significant issue in bronchiectasis and NTM infection^27^. Treatment with high doses of ICS is associated with significant risk of systemic side effects such as adrenal suppression, development of diabetes and of osteoporosis. Therefore, it is crucial that they are only used where there is a clear indication.

If a patient is suspected of having bronchiectasis without asthma or COPD, they should not be started on ICS^7^. Whilst this is straight forward for a patient presenting with classical symptoms of bronchiectasis, it is more complicated for those with suspected or confirmed asthma or COPD who have been initiated on ICS treatment^48^. Some patients will have co-morbid asthma or COPD alongside bronchiectasis. This overlap between airways disease can make it difficult to decide whether an ICS should be prescribed. In a patient with possible bronchiectasis who has a prior diagnosis of asthma or COPD, it is important to review those diagnoses to establish whether they are accurate.

It is useful to find out when the diagnosis was made, by whom and with what supporting evidence. UK guidelines^49^ recommend that asthma should be diagnosed using objective testing where possible. However, in primary care, access to spirometry, peak flow monitoring, or FeNO testing may be limited or delayed. International guidance, including the Global Initiative for Asthma (GINA)^50^, recognises this and allows for a trial of treatment in patients with a high clinical probability of asthma when objective testing is unavailable, with confirmation of the diagnosis at a later stage. In patients whose clinical history is not suggestive of asthma, who have never had objective evidence to support the diagnosis, and who have not responded to inhaled corticosteroids used at appropriate doses with correct technique, a diagnosis of asthma is unlikely.

The diagnosis of COPD requires a relevant exposure history, respiratory symptoms, and spirometry showing airflow obstruction. This typically includes a smoking history of 10 pack-years or more, or another exposure such as biomass fuel, with a post-bronchodilator FEV₁/FVC ratio of <0.7 or below the lower limit of normal^37^.It is well established that diagnoses of COPD are sometimes made without supporting spirometry, as was the case during COVID when no spirometry was available. Patients diagnosed with COPD without prior spirometry should have spirometry arranged to clarify the diagnosis^11^. If patients have airflow obstruction but have never smoked and have no other relevant exposure, this should not result in a label of COPD, as airflow obstruction is common in bronchiectasis^16^. Given the overlap between COPD and bronchiectasis, the “ROSE” criteria can be useful^11^. The ROSE criteria state that a diagnosis of COPD can be made in the presence of bronchiectasis if there is a smoking history of at least 10 pack-years (or another relevant exposure such as biomass fuel) and a postbronchodilator FEV1/FVC of <0.7.

Avoiding mislabelling respiratory disease helps prevent inappropriate use of ICS and reduces exposure to potential side effects without clinical benefit. Breathlessness is common in people with bronchiectasis, and a trial of dual bronchodilator (long acting beta agonist and long acting muscarinic antagonist (LABA/LAMA)) treatment is reasonable in patients with significant breathlessness^51^.

**Management of acute exacerbations**

**Fig. 3:**
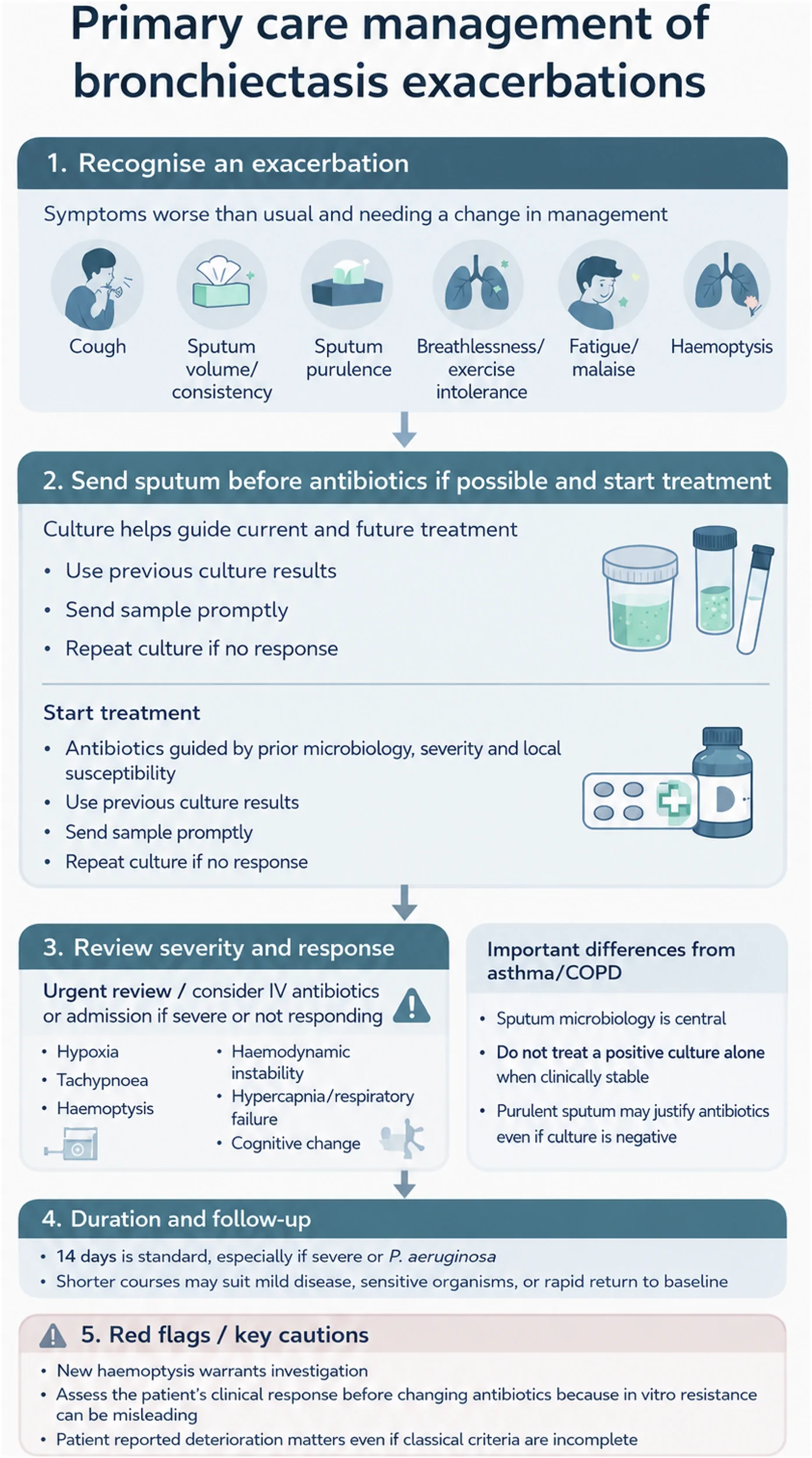
Management of exacerbations in primary care (Image created with AI assistance).

Primary care has a critical role in the management of exacerbations and most of the recommendations in this section of the guidelines are highly relevant to those working in primary care. There are key differences in the management of bronchiectasis exacerbations compared with those of asthma and COPD. Figure 3, below, summarises management of exacerbation.

Bronchiectasis exacerbations are heterogeneous in their presentation and may not always conform to predefined criteria. Patients frequently recognise early or subtle changes in their baseline symptoms, and clinician assessment should take patient-reported deterioration into account, even when classical features are not yet present.

Oral corticosteroids are not routinely used in the management of bronchiectasis exacerbations, unless the patient also has asthma or COPD and is displaying signs of bronchoconstriction (chest tightness, wheeze). In patients who have overlapping asthma or COPD who often get both antibiotics and steroid treatment prescribed, it can be useful to give each treatment individually to assess which component is helpful (e.g. treat with antibiotics for 48 h then review symptoms to determine if oral steroid treatment is needed).

Rescue packs can be valuable for patients to allow them to access prompt treatment for an exacerbation without waiting to speak to a clinician, then waiting for the prescription to become available. Rescue pack prescriptions require that the clinician is confident that the patient can identify an exacerbation, that the treatment that works for the patient is clear (e.g. antibiotics or antibiotics and steroids) and that the patient is happy to let the clinician know that they have commenced the treatment, so that exacerbation frequency can be easily monitored.

Definition and investigation^7^:

- An exacerbation is defined as a worsening of symptoms that exceeds day-to-day variability and requires a change in management. Core symptoms of exacerbation include a change in cough, sputum volume and/or consistency, sputum purulence, dyspnoea and/or exercise intolerance, fatigue or malaise and haemoptysis. Additional clinical features are fever, wheeze, general discomfort, anorexia, weight loss, pleuritic chest pain, and changes on chest examination^52^.
- Features of a severe exacerbation (defined as requiring hospitalization or intravenous antibiotic treatment) may include tachypnoea, acute or acute on chronic respiratory failure, a significant decline in oxygen saturation or respiratory function, hypercapnia, haemoptysis, new onset of cyanosis, new signs of *cor pulmonale*, hemodynamic instability, and/or impaired cognitive function. IV antibiotic treatment may also be used for resistant organisms or in patients who have not responded to oral antibiotic treatment.

- At the onset of an exacerbation, a sputum sample for microbiology should ideally be obtained before initiating antibiotic treatment. Samples should reach the laboratory promptly to avoid false negative results.
- Sputum culture should be repeated if there is no response to the initial antibiotic treatment.

Treatment:

- Antibiotics should be prescribed for an exacerbation, guided by previous microbiology results, local susceptibility patterns, and clinical severity.
- An adult bronchiectasis self-management plan should include guidance on recognising exacerbations. Providing selected patients, the ability to self-administer antibiotics at home with appropriate instruction and education, may allow more prompt treatment.
- Patients not responding promptly to oral antibiotics or showing signs of a severe exacerbation, should be reviewed to determine if there is a need for a change in treatment, intravenous antibiotic treatment, and/or hospitalization.
- Airway clearance regimens may need to be adapted in frequency, intensity, and technique during an exacerbation.
- In general, a 14-day antibiotic course is considered standard, especially in severe exacerbations or in patients with *P. aeruginosa infection*. Shorter courses may be appropriate in patients with mild bronchiectasis, those with infection due to pathogens more sensitive to antibiotics (e.g. *S. pneumoniae*), or patients with a rapid return to baseline symptoms during treatment.

Sputum samples are valuable in guiding treatment, but they have some important limitationsSamples often show no growth of organisms, particularly if the patient has recently received antibiotics, or if there has been a delay in transport to the laboratory. Because sputum culture has limited sensitivity, a negative result does not exclude the need for antibiotics, particularly when sputum is purulent. Purulent sputum is not normal and reflects the presence of neutrophils, which are often associated with the presence of bacterial infection^2,53^. Conversely, it is common for patients with bronchiectasis to grow pathogens when clinically stable. In the absence of a deterioration in symptoms, a positive sputum culture alone should not prompt antibiotic treatment.

Antibiotic resistance testing is useful but can be misleading. If resistance is reported several days after treatment has started, clinical response should be assessed. Treatment should only be changed if the patient is not showing clinical response to the current antibiotic. In the situation where the organism grown is sensitive to the antibiotic given, but after 7–10 days the patient is not showing any improvement, a change in therapy may be required. In the absence of asthma or COPD, oral corticosteroids generally are not indicated in the management of bronchiectasis exacerbations.

Haemoptysis can be the main symptom of an exacerbation. Haemoptysis is usually treated with antibiotics in bronchiectasis patients but is a red flag symptom and,if new, should prompt further investigation such as x-ray, and discussion with secondary care who may recommend further chest imaging such as CT.

### Recognising and managing the deteriorating patient

Bronchiectasis is a condition that may deteriorate slowly or rapidly over time or remain stable for many years. A common presenting situation in primary care is the deteriorating patient. In the ERS 2025 guidelines, deterioration is defined by increasing exacerbation frequency and/or severity, worsening symptoms, and/or a rapid decline in lung function^7^.

**Fig. 4:**
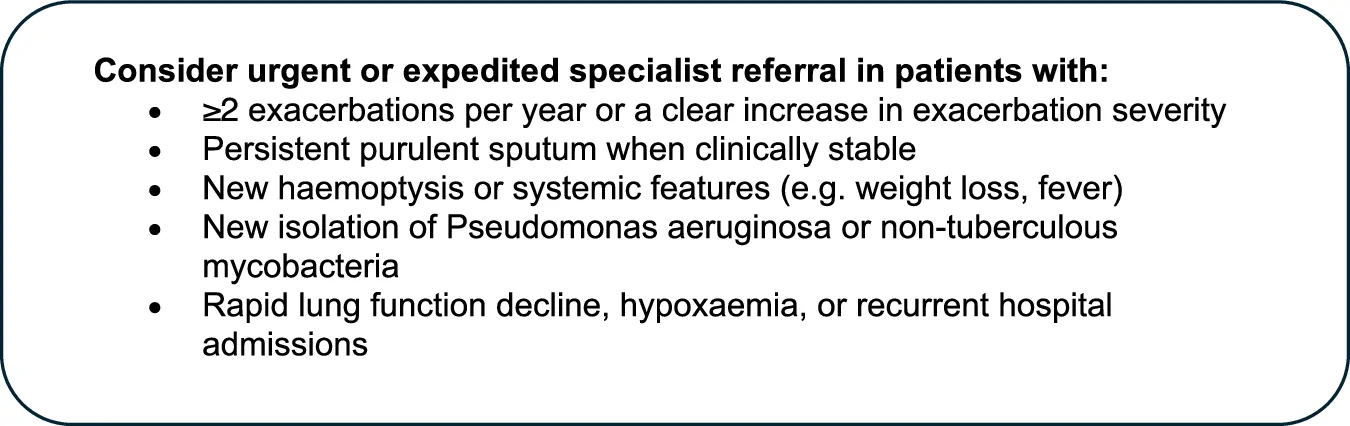
when to consider urgent/expedited referral to respiratory specialist.

Patients with bronchiectasis who present with a persistent change in symptoms compared with their baseline, or with repeated or increasingly severe exacerbations, should be reviewed and referred to their respiratory specialist. Figure 4 shows clinical features that would suggest a referral to the respiratory specialist should be considered. Important components of a review in this situation would include:

- an up to date history to check for the development of any new co-morbidities or other significant diagnoses (pneumonia, pulmonary embolism, lung cancer, heart disease)
- review the management of existing co-morbidities
- a discussion with the patient around adherence with treatments.
- A chest x-ray, full blood count, c-reactive protein, and a sputum for culture including NTM are reasonable tests for primary care professionals to perform prior to referring the patient to secondary care. As noted above it is important that samples reach the laboratory as soon as possible after expectoration.
- Where spirometry testing is available in a clinically relevant time frame, this could also be performed.
- Review of mental health; deterioration is distressing for patients and will heighten psychosocial challenges including the stigma associated with coughing and the impact this has on social connections. Discuss with the patient and offer support as available locally. This may include support groups, counselling and therapy.

## Summary

Bronchiectasis is a common disease with a high impact on patients’ quality of life and has an increasing prevalence and newly licensed treatments becoming available. The 2025 ERS clinical practice guidelines for the management of adult bronchiectasis is highly relevant to primary care professionals involved in the diagnosis and management of bronchiectasis patients. Table 2 summarises the key messages.

**Table 2:** Summary of important topics and key recommendations with relevance to primary care.

Recommendation	Key points for primary care
**Suspect and diagnose bronchiectasis**	● Suspect in patients with persistent cough, sputum production or recurrent chest infections ● Bronchiectasis may complicate COPD or asthma so suspect bronchiectasis in these patients particularly if management is not straightforward ● Patients with other inflammatory conditions (IBD, RA, CTD) are at increased risk of bronchiectasis and a higher index of suspicion should be held in these patients.
**Assessing newly diagnosed bronchiectasis (a combination of primary and secondary care activities depending on the system organisation)**	● Patients suspected of having bronchiectasis should have blood tests for: Full blood count, Immunoglobulins, serum protein electrophoresis, ABPA serology and sputum sent for culture, including NTM. ● Important co-morbidities should be identified and managed.Patients with more than 2 exacerbations in a year, increasingly severe symptoms, persistently purulent sputum, those with high risk causes and those growing pseudomonas or NTM in their sputum should be referred for urgent consultant review.
Airway clearance and mucoactives	● Refer patients for airway clearance training with a specialist respiratory physiotherapist. ● Mucoactives are best started in secondary care.
Pulmonary rehabilitation	● Bronchiectasis patients benefit from pulmonary rehabilitation. ● Identify and refer those starting to experience functional limitation and those with established functional limitations.
Long term antibiotics	● Long term oral and inhaled antibiotics are started in secondary care. ● Gastrointestinal side effects are common with long term macrolides. Hearing loss and tinnitus are rare but important side effects. ● Inhaled antibiotics can cause bronchospasm
Pseudomonas eradication	● Any new isolation of Pseudomonas in sputum should prompt discussion with secondary care
Inhaled corticosteroids	● Inhaled corticosteroids are not indicated in the management of bronchiectasis without the presence of asthma or COPD with indication for ICS
Acute exacerbations	● Acknowledge heterogeneity in exacerbation symptoms – listen to your patient. ● Send sputum for culture, ideally before starting antibiotics. ● Start empirical antibiotic therapy, guided by prior culture if available. ● Check for signs of severe exacerbations. ● Treat new haemoptysis as a red flag requiring further investigation ● Advice to patient regarding self-management including increased airway clearance, concordance with antibiotic treatment and good hydration. ● Safety netting and worsening advice should be given.
The deteriorating patient	● Deteriorating patients should be referred for urgent consultant review.

## Data Availability

Not applicable
